# Retrospective observational study applying reinforcement learning and behavioral science to mammography: insights for equitable preventive care

**DOI:** 10.1093/abm/kaag024

**Published:** 2026-05-29

**Authors:** Emily Frith, Sarah Deedat

**Affiliations:** Behavioral Reinforcement Learning Lab (BReLL), Lirio, Inc., 320 Corporate Drive, NW, Knoxville, TN 37923, United States; Behavioral Reinforcement Learning Lab (BReLL), Lirio, Inc., 320 Corporate Drive, NW, Knoxville, TN 37923, United States

**Keywords:** artificial intelligence, digital health, mammography, personalized medicine, health equity

## Abstract

**Background:**

In the United States, underserved women face persistent healthcare disparities, making mammography screening and equity public health priorities.

**Purpose:**

This retrospective observational study assessed how tailored messaging influenced mammogram scheduling and attendance, determined which message types were most effective, and explored differences across key demographic subgroups.

**Methods:**

We employed Precision Nudging using reinforcement learning and behavioral science to tailor email messages informed by the Capability, Opportunity—Behavior (COM-B) model. Messages were sent to 156 954 women overdue for screening. Mammogram scheduling and attendance were documented via electronic health records. Analyses focused on the engaged patient population who opened at least one email (*N* = 106 616; 67.9%). Logistic regressions examined whether COM-B content in the first and last message predicted scheduling and attendance, testing interactions for age, race, income, insurance, and education.

**Results:**

Among engaged patients, 23.7% scheduled and 20.3% attended a mammogram. Capability-focused messaging was generally associated with higher overall uptake, while opportunity messaging consistently predicted lower scheduling and attendance rates. Equity analyses revealed nuanced engagement patterns among underserved groups. Underrepresented racial/ethnic minority women had higher odds of completing screening than White women. Older age predicted lower attendance, with early messages addressing motivation predicting lower attendance than capability messages. Final intervention messages addressing motivation were more effective for low- and high-income women than middle-income women and more effective for women without private insurance.

**Conclusions:**

These findings suggest that tailoring messages to patients’ needs and context encourages mammogram uptake and may help reduce disparities in women’s health.

## Teaser

Tailored email messages were sent using artificial intelligence to encourage overdue women to schedule and complete recommended mammograms. The message type that was most helpful differed by age, race, income, insurance coverage, and education.

## Introduction

The US Preventive Services Task Force recommends biennial mammography for all women ages 40-74^1^ for early detection and diagnosis of breast cancer, which is generally more treatable in early stages. According to the American Cancer Society, an estimated 49% of women aged 40 years or older were screened within the past year, and 67% were screened within the past 2 years,^2^ highlighting a gap between clinical recommendations and real-world adherence. Screening prevalence varies considerably in the United States, with approximately 1 in 5 women overdue for a recommended mammogram.^3^

Two objectives of Healthy People 2030 are to increase the proportion of females who receive a breast cancer screening^4^ and to reduce breast cancer mortality.^5^ Both screening and mortality rates are improving, but healthcare disparities remain a persistent obstacle to women’s health. Socioeconomic and racial inequities contribute to gaps in screening and diagnoses, which can lead to increased mortality rates in underserved women.^6^ Interventions that reduce structural barriers to mammogram screening were recommended by the Community Preventive Services Task Force to increase equitable access to screening services.^7^ Example strategies for reducing structural barriers to obtaining mammograms include mitigating time constraints, such as modifying service hours to meet patient needs and simplifying patient scheduling and healthcare portal navigation.

Digital Health Interventions (DHIs) offer a scalable approach to mammography participation, particularly among historically underserved populations. Advances in artificial intelligence (AI), machine learning, telemedicine, and big data have led to profound changes in healthcare delivery, allowing for widespread patient engagement beyond traditional clinical encounters.^8^ Strategic application of these technologies can better engage patients in their health decisions and expand equitable, timely access to care. We evaluated an email-based DHI that used tailored reinforcement learning and behavioral science strategies informed by the Capability, Opportunity, Motivation—Behavior (COM-B) model to encourage breast cancer screening in women overdue for care.

The COM-B model is a widely used framework for behavioral intervention design because it accounts for multiple interacting influences on health behavior.^9^ This model posits that a health behavior, such as getting a mammogram, will occur when an individual has (1) the physical skills and psychological *capability* to engage in the behavior; (2) an *opportunity* to act, afforded by physical and social factors in the external environment; and (3) reflective (eg, evaluation and planning) and automatic (eg, impulses and emotions) *motivation* to perform that specific behavior over other competing behaviors.^9^^,^^10^ The COM-B model has been used to evaluate cervical cancer screening behaviors and barriers to attendance,^11^^,^^12^ nutrition and weight management among breast cancer survivors,^13^^,^^14^ cancer patients’ involvement in treatment decision-making^15^ and public attitudes toward genomic screening for prostate and breast cancer.^16^ Additionally, a pilot study from our group demonstrated that tailored DHIs are feasible for engaging overdue patients across demographic subgroups to schedule and attend recommended screenings, indicating potential for promoting screening equity in diverse patient populations.^17^ However, there is no scientific evidence to date establishing whether tailored, COM-B-informed DHIs encouraging breast cancer screening in real-world health systems or which patient populations are responsive to specific intervention components.

This DHI was informed by both the COM-B model and reinforcement learning, a form of AI that iteratively selects and delivers messages based on observed patient behavior.^18^ Initially, the reinforcement learning algorithm used existing patient data (eg, age, race, gender, last mammogram screening, etc.), which were automatically captured from medical records, to determine message selection. Subsequent message engagement (eg, message opens, clicking links to schedule, and mammogram attendance) served as a reinforcement signal to adapt the delivery of specific COM-B-informed message types based on downstream behavioral rewards, rather than explicit patient feedback. As data-driven knowledge accumulates, the system can better estimate which behavioral science strategies are more likely to encourage screening among patient subgroups that share similar characteristics and response patterns. The primary objectives of this research were to evaluate whether messaging informed by the COM-B model promoted mammogram scheduling and attendance among women overdue for screening, to identify the most effective message types, and to determine whether messaging effects differed across key demographic subgroups.

## Methods

### Participants and setting

This research was approved by a private institutional review board accredited by the Association for the Accreditation of Human Research Protections Programs. The DHI was delivered via email to 156 954 women (mean [X̄] age: 61.9 years; range: 44-80 years) from August 2020 to December 2024. Participants were patients in 2 US health systems who were overdue for a mammogram (≥18 months since last mammogram for health system 1, or ≥24 months since last mammogram for health system 2) and had opted to receive health-related communications. Eligibility criteria and overdue status were determined by each health system’s internal clinical requirements, risk assessments, and outreach protocols. Each health system provided population-level electronic medical record (EMR) data to support eligibility determination and data integration, including providing daily updates on mammogram scheduling and attendance, changes in age, health status, and other factors affecting eligibility. Patients were dynamically added to or removed from the intervention distribution list as their eligibility status changed (eg, reached overdue status: eligible: received a screening elsewhere: ineligible, etc.).

Demographic variables included age, race, income, insurance, and educational attainment. For model stability and interpretability, race was dichotomized as white or underrepresented groups (Black or African American, Asian/Pacific Islander, Hispanic, American Indian/Alaska Native, multiracial, or other). Annual household income was categorized as low (<$30 000), middle ($30 000-$74 999), or high (≥$75 000). Insurance was classified as commercial (private insurance through an employer or private insurance company) or noncommercial (Medicare, Medicaid, ­military coverage, ChampVA, worker’s compensation, self-pay/no insurance, or other non-private insurance.) Due to the level of access to patient insurance data, measurement constraints did not permit separation of self-pay from other forms of noncommercial coverage; therefore, noncommercial insurance consists of both uninsured (self-pay) and publicly insured women. Educational attainment was categorized as high school or technical school, undergraduate, or postgraduate.

### Email content design and send schedule

Breast cancer screening outcomes included scheduling and attending a mammogram. Barriers to achieving these outcomes were identified through a review of published studies. Two behavioral scientists reviewed the identified determinants, mapped each barrier to its underlying mechanism of action, and linked these mechanisms to relevant behavior change techniques using the Theory and Techniques Tool.^19^ Emails were then developed using behavioral science strategies to address each barrier. Table 1 provides examples of barrier categorization, strategies to reduce barriers, corresponding approaches to email content design, and examples of emails sent during this DHI. Each email was linked to 1 specific COM-B component. Given that AI-based reinforcement learning was used to personalize email selection and timing to address specific barriers related to capability, opportunity, or motivation based on individual characteristics, the distribution of messages received by each patient varied by design. Specifically, the reinforcement learning algorithm tailored the selection of human-designed email messages to deliver by generating unique vector embeddings of each patient’s characteristics (eg, prior engagement patterns, demographics, etc.), comparing these to embeddings of each message’s content and tone, and selecting the email expected to maximize behavioral outcomes. Over time, observed responses, including opens, scheduling, and attendance, further refined message selection. Message send time was also informed by historical engagement patterns (day of week, time of day, etc.) and patient characteristics to identify the days and times that each patient was most likely to respond positively to outreach. Over time, the algorithm learned which messages tend to lead to favorable outcomes based on accumulated reward signals. For example, higher reward values were assigned to actions most proximal to mammogram scheduling and attendance, with fewer points assigned to earlier engagement indicators such as message opens and link clicks. Unsubscribes were assigned a negative reward value. The reward function refined tailoring over the course of the intervention by continuously updating the algorithm’s predictions about which message and send time would be most likely to nudge a patient toward action.

**Table 1:** Examples of the email content design process.

Barriers to mammogram scheduling and attendance	COM-B category	Behavioral science strategy to reduce barriers	Summary approach for applying strategy in email content	Example email
**Knowledge and awareness**	Capability	Provide information about health consequences	Emphasizes health consequences of delayed or missed mammography (eg, reduced likelihood of early detection) Provides information on breast cancer risk factors and common symptoms to address knowledge gaps and increase screening awareness	[FirstName], do you know your risk? About 1 in 8 women will develop breast cancer in her lifetime. Your risk may go up with age, even if you don’t have a family history. That’s why your regular mammogram is so important. It can show changes in your breasts *before* they become a problem—which can give you more control over your health. Schedule your mammogram today at [ClinicName].
**Self-efficacy**	Capability	Verbal persuasion about capability	Reinforces patient capability to schedule and complete a mammogram by reducing perceived friction related to appointment logistics and visit navigation	[FirstName], you’ve got this. We all doubt ourselves sometimes. But we can’t let doubt stand in the way of our well-being, right? You have what it takes to protect your health by getting your mammogram. A mammogram is a simple screening with a major impact on your health and your life. Think about how great you’ll feel once you’ve taken the first step. Why not schedule your appointment today?
**Clinical/professional factors**	Opportunity	Credible source	Highlights recommendations from trusted clinical and public health authorities to support screening uptake	Feeling nervous? You’re not alone. Plenty of women feel the same way about mammograms. But believe it or not, they’re always glad they went. The reassurance of knowing they’re taking care of their health is worth any discomfort. Your [ProviderNames] deal with all sorts of people and different types of bodies every day. They’re trained to be professional and respectful, and they understand how you feel. Mammograms are quick and private. So, there’s no need to feel uncomfortable or embarrassed. Be brave, take the first step, and schedule your appointment.
**Social support**	Opportunity	Social comparison	Highlights how similar women (“women like you”) prioritize staying current on breast cancer preventive care, emphasizing descriptive social norms rather than social approval	Join the movement. Most women your age are staying up to date with their mammograms. Are you? Your friends, family members, and neighbors are taking action to catch breast cancer early. Join them by scheduling your mammogram, then spread the word and help other women stay healthy, too. Call or click below to schedule your appointment now.
**Fear**	Motivation	Reduce negative emotions	Addresses fears of discrimination by emphasizing equitable, supportive care Normalizes anxiety and reassures patients about comfort, safety, and respectful treatment during	Ouch! What if it hurts? If you’re worried that your mammogram may hurt, you’re not alone. While it can be a bit uncomfortable, rest assured that any pain is temporary. And your future health is worth it. Ask your [ProviderName] if it’s safe to take over-the-counter pain medicine before your appointment. And mention your concerns to staff at the imaging center—there are steps they can take to minimize your discomfort. Ready to put your mind at ease? Schedule your appointment.
**Psychological factors**	Motivation	Identification of self as role model	Frames mammogram attendance as an opportunity to serve as a role model for other women	[FirstName], be a role model for the women you love. It starts with getting your regular mammogram. We know this visit may not be your favorite thing to do. But showing up sets a good example. Are you willing to be a role model for the amazing women in your life? If so, schedule your mammogram now.

Abbreviation: COM-B, Capability, Opportunity—Behavior.

This table summarizes the scientific approach used to design email content, with example messages.

One weekly email was sent to each patient for 5 weeks, followed by an 8-week pause to help counteract notification fatigue; this cadence repeated until the patient either attended a mammogram, unsubscribed from intervention-related messages, or the intervention concluded. Over the study period, patients received up to 43 emails (X̄ = 14).

### Analysis

We measured 2 primary binary outcomes for each patient over the intervention period: (1) patient scheduled a mammogram appointment and (2) patient attended a mammogram (completed the screening). Patients who scheduled but had not yet attended by study end were counted in the scheduling outcome only. To quantify the true effects of intervention exposure, we focused analyses on patients who opened/clicked at least 1 email (hereafter referred to as “engaged”), as exposure could not be assumed for non-openers.

Logistic regressions were conducted among engaged patients to examine whether COM-B content in the first or last message predicted mammogram scheduling and attendance. Message effects were evaluated at these consistent and theoretically relevant anchors in the behavioral decision process because the initial message not only introduced participants to the mammogram-focused DHI, but also likely shaped cognitive and behavioral responses to subsequent messages. The final message was most proximal to action and represented the most recent opportunity to influence scheduling and attendance decisions. In both the first and last email models, the main independent variable was the first/last email’s COM-B content, which was a 3-level factor variable with capability as the reference category. Given variability in the total number of messages sent to patients, we controlled for message volume in the last email models. We also controlled for single-message exposures because, for individuals who only received 1 email, their “first” and “last” email was the same. This approach isolated the effect of content exposure from the number of contact attempts. We followed up logistic regressions with interaction models to evaluate moderation effects of demographic characteristics on mammogram attendance. Odds ratios (ORs) with 95% CIs and/or *P-*value are reported for regression outcomes below. Statistical significance was set at *P* < .05 (2-tailed). All data were analyzed using RStudio Version 2025.09.2 + 418 (2025.09.2 + 418).

## Results

### Participant characteristics

Over 2/3 of participants engaged with (ie, opened/clicked) one or more intervention emails (*N *= 106 616, 67.9%). About 1/3 did not engage with any email content (*N* = 50 338; 32.1%), and 0.48% (*N* = 754) unsubscribed from intervention emails. Overall, 23.7% (*N* = 25 260) of engaged participants scheduled a mammogram, and 20.3% (*N* = 21 639) attended a mammogram. Among engaged patients with a verified prior mammogram date (*n* = 32 983), each additional year since the last mammogram was associated with lower odds of scheduling (OR = 0.78 per year) and attendance (OR = 0.79 per year; both *P*s < .001). Non-missing demographic data for the overall, engaged, and unengaged populations and comparisons between engaged and unengaged patients are described in Table 2. The average age of women in all 3 populations was between 61 and 62 years. Most women had a high school/technical school education and were middle-income, White, and not commercially insured.

**Table 2:** Demographic characteristics and comparisons between engaged and unengaged samples.

Variable	Overall sample *N =* 156 954	Engaged sample (opened ≥1 email) *N* = 106 616	Unengaged sample (never opened an email) *N* = 50 338	Test statistic (df), *P*-value Engaged vs. unengaged *N* = 156 954
**Age**	97 636 (61.88-7.80)^a^	70 528 (62.06-7.47)	27 108 (61.41-8.59)	*t *= −10.93 (df = 43 752), *P* < .001^b^
**Income**				χ² = 265.18 (df = 2), *P* < .001
**Low**	12 812 (17%)^c^	9622 (16.3%)	3190 (19.5%)	
**Middle**	33 285 (44.2%)	25 611 (43.5%)	7674 (47.0%)	
**High**	29 165 (38.8%)	23 704 (40.2%)	5461 (33.5%)	
**Education**				χ² = 48.99 (df = 2), *P* < .001
**High school or technical school**	32 889 (49%)^c^	25 485 (48.4%)	7404 (51.1%)	
**Undergraduate education**	24 455 (36.4%)	19 280 (36.6%)	5175 (35.7%)	
**Postgraduate education**	9842 (14.6%)	7940 (15.1%)	1902 (13.1%)	
**Insurance**				χ² = 1525.2 (df = 1), *P* < .001
**Commercially insured**	41 481 (42.5%)	32 666 (46.3%)	8815 (32.5%)	
**Not commercially insured**	56 155 (57.5%)	37 862 (53.7%)	18 293 (67.5%)	
**Race**				χ² = 16.46 (df = 1), *P* < .001
**White**	85 413 (87.5%)	61 887 (87.7%)	23 526 (86.8%)	
**Underrepresented**	12 223 (12.5%)	8641 (12.3%)	3582 (13.2%)	
**Last mammogram**				χ² = 1015.08 (df = 2), *P* < .001^d^
**≥18 months to 2 years (Health System 1 only)**	2314 (1.5%)	995 (0.9%)	1319 (2.6%)	
**>2-3 years (Health Systems 1 and 2)**	26 960 (17.2%	19 370 (18.2%)	7590 (15.1%)	
**>3 years (Health Systems 1 and 2)**	16 864 (10.7%)	12 618 (11.8%)	4246 (8.4%)	
**No prior documentation**	110 816 (70.6%)	73 633 (69.1%)	37 183 (73.9%)	

a *n* (mean, SD) using non-missing age data;.

b Statistical comparisons between engaged and unengaged groups using non-missing data.

c *n* (%) using non-missing data for categorical variables.

d χ^2^ test was computed among patients with a verified prior mammogram record only (*n* = 46 138; categories: ≥18 months to 2 years, >2-3 years, >3 years). Patients without prior documentation were excluded from the test as these patients have no screening on file.

### Messaging outcomes

#### First message

Among engaged participants, the first email message most often addressed motivation (*n* = 69 889), followed by capability (*n* = 35 201) and opportunity (*n* = 1526). Unadjusted scheduling rates were highest when capability content was messaged first (24.3%), followed by motivation (23.5%) and opportunity messages (20%). Unadjusted attendance rates were lower overall but followed a similar pattern: 20.4% for capability or motivation ­content and 16.1% for opportunity.

We next estimated logistic regression models to test whether observed differences in scheduling and attendance varied by the COM-B category targeted in the first message (reference: capability). Motivation-targeted messages were associated with lower odds of scheduling (OR = 0.958; 95% CI: 0.929-0.987), as were opportunity-targeted messages (OR = 0.779; 95% CI: 0.685-0.884). For attendance, motivation did not differ from capability (OR = 1.000, 95% CI: 0.969-1.033), whereas opportunity messages were associated with lower odds of attendance (OR = 0.752; 95% CI: 0.653-0.862). Pairwise contrasts using Tukey adjustment for multiple comparisons confirmed that motivation and capability were significantly more effective than opportunity for both outcomes (ORs = 1.23-1.33, *P*s ≤ .004).

#### Last message

Among engaged participants, the last email message most often addressed capability (*n* = 56 738), followed by motivation (*n* = 34 172) and opportunity (*n *= 15 706). Unadjusted scheduling rates were highest for motivation (29.5%), followed by capability (23.6%) and opportunity (11.5%). Unadjusted attendance rates were lower than scheduling, but with a similar pattern: 27.7% for motivation, 19.2% for capability, and 8% for opportunity content (see Table 3).

**Table 3:** Direction of COM-B messaging effects on scheduling and attendance by message position.

Outcome	Message position	COM-B comparison		
		Capability vs. motivation	Capability vs opportunity	Opportunity vs. motivation
**Scheduling**	First	↑	↑	↓
	Last	↑	↑	↓
**Attendance**	First	—	↑	↓
	Last	↑	↑	—

Abbreviation: COM-B, Capability, Opportunity—Behavior.

Arrows reflect adjusted logistic regression estimates. Upward arrows (↑) indicate that the first message type listed was associated with higher odds of the outcome vs. the second, downward arrows (↓) indicate lower odds, and dashes (—) indicate no significant difference.

We conducted logistic regression models to predict scheduling and attendance from the COM-B category targeted in the last message (reference: capability), adjusting for the number of messages received (mean-centered) and single-message exposures (binary indicator). Following adjustment, motivation-targeted messages were associated with lower odds of scheduling and attending (scheduling OR = 0.691, 95% CI: 0.667-0.715; attending OR = 0.755; 95% CI: 0.728, 0.783), as were opportunity-targeted messages (scheduling OR = 0.805, 95% CI: 0.760-0.852; attending OR = 0.786; 95% CI: 0.735, 0.839).

Each additional message received predicted lower odds of scheduling and attending (ORs ≤ 0.902, *P*s < .001), and single-message participants also had lower odds of scheduling (ORs ≤ 0.728, *P*s < .001). Tukey-adjusted pairwise contrasts indicated that motivation messages were associated with higher odds of scheduling than opportunity messages (OR = 0.859, *P* < .001) but were not significantly different from opportunity for attendance (OR = 0.961, *P* = .520). Table 3 summarizes the direction of each COM-B comparison for scheduling and attendance. In the first and last message models.

### Health equity results

Separate logistic regression models were fit to examine whether age, race, income, insurance, and education moderated the relationship between COM-B content in the first and last intervention messages and mammogram attendance. To reduce redundancy, only significant main effects and interactions are described in the text; significant message effects, subgroup effects, and interaction terms are also presented in Table 4, with more comprehensive model results reported in Table S1. Table 5 provides a high-level summary of key findings and implications of the outcomes discussed below.

**Table 4:** Health equity logistic regression model results.

Model	Main demographic effects (OR [95% CI])	COM-B comparison effects (ORs, *P*-values)	Significant interactions
**First message**	Income: lower odds of attendance for low vs. middle (OR: 0.757 [0.589-0.962]) and for high vs. middle (OR: 0.817, [0.683-0.975]) Insurance: lower odds for noncommercial (OR: 0.544, [0.474-0.622]) vs, commercial	Motivation: higher odds of attendance across all groups vs. capability (all ORs: 1.288-1.338, all *P*-values <.03) Opportunity: higher odds for age, income, education vs. Capability (ORs: 1.309-2.140, p-values <.04	Age × motivation: motivation-first messages associated with lower odds of attendance with older age (OR: 0.985 [0.975-0.995])
**Last message**	Age: lower odds of attendance with older age (OR: 0.986 [0.979-0.995]) Race: higher odds for underrepresented vs. White (OR: 1.474 [1.218-1.773]) Income: lower odds for high vs. middle (OR: 0.545 [0.459-0.645]) and higher odds for low vs. high (OR: 1.996 [1.667-2.391]) High school: higher odds for high school (OR: 1.609 [1.267-2.064]) and for undergraduate (OR: 1.331 [1.036-1.724]) vs. postgraduate	Motivation: higher odds of attendance for age, race, income, and education vs. capability (ORs: 1.196-1.722, *P*-values <.001) Opportunity: higher odds across all groups vs. capability (ORs = 1.741-2.434, *P*-values <.001) and ↑ attendance for age, race, and insurance vs. motivation (ORs = 0.532-0.607, *P*-values <.001)	Income × motivation: motivation-last messages associated with higher odds of attendance for low-income (OR: 1.347 [1.013-1.795]) and high income vs. middle income (OR: 1.439 [1.162-1.784]) Income × opportunity: opportunity-last messages associated with higher odds of attendance for high income vs. middle income (OR: 1.530 [1.070-2.185]) Insurance × motivation: Motivation-last messages associated with higher odds of attendance for noncommercial vs. commercial insurance (OR: 1.865 [1.577-2.205])

Abbreviations: COM-B, Capability, Opportunity—Behavior; OR(s), odds ratio(s).

**Table 5:** Key outcomes and implications of the health equity analyses.

Demographic	Key findings	Messaging implications
**Age**	Motivation-first messages decreased odds of attending by ∼1.5% with each year over age 62. Older age was related to lower attendance following the last message.	Motivation and opportunity messaging may be more effective for older women, with motivation messaging becoming less encouraging with age.
**Race**	Underrepresented women had higher odds of attending than White women across message types.	Motivation and opportunity messaging were consistently more effective than capability messaging.
**Income**	Low-income women were less likely to attend than middle-income women following the first message, but more likely to attend than high-income women following the final message	Motivation-last messages may be particularly impactful for low- and high-income women; opportunity framing may also facilitate attendance in high-income women.
**Insurance**	Non-commercially insured women were overall less likely to attend screenings.	Motivation-first and opportunity-last sequencing were broadly encouraging, but a final motivational message may be more effective for non-commercially insured women.
**Education**	Postgraduate education was associated with lower odds of attendance	Motivation and opportunity messages were consistently stronger than capability messages across education levels.

#### Age

Age was negatively associated with attendance in the last message model (OR = 0.986, *P* < .001). A significant age × motivation interaction (OR = 0.985, *P* = .003) indicated that the effectiveness of motivation-first messages decreased by about 1.5% with each additional year above the mean age (62.06).

#### Race

Underrepresented women had higher odds of attending than White women, averaged across message content (OR = 1.474, *P* < .001).

#### Income

Low-income women had significantly lower odds of attendance compared to middle-income women (low OR = 0.757, *P* = .026) in the first message model. High-income women had significantly lower odds of attendance compared to middle-income women in both models (first OR = 0.817, *P* = .026; last OR = 0.545; *P* < .001). In the last message model, low-income women had approximately double the odds of attendance compared with high-income women (OR = 1.996, *P* < .001).

Significant interactions emerged in the last message model, indicating that motivation messages were more effective for low-income women (OR = 1.347, *P* = .041) and high-income women (OR = 1.439, *P* < .001) than for middle-income women, and opportunity messages were more effective for high- versus middle-income participants (OR = 1.530, *P* = .019).

Follow-up contrasts showed that capability messages were less effective than motivation messages across all income groups (ORs = 0.531-0.764, *P*s ≤ .0007) and were less effective than opportunity messages for middle-income women (OR = 0.375, *P* < .001) and high-income women (OR = 0.574, *P* < .001). Motivation messages were also less effective than opportunity messages in high-income women (OR = 0.707, *P* = .0231).

#### Insurance

Non-commercially insured women (ie, self-pay or not privately insured) had significantly lower odds of attendance compared to women with commercial (ie, private) insurance coverage in the first message model (OR = 0.544; *P* < .001). One significant interaction indicated that motivation-last messages predicted higher odds of attendance for non-commercially insured women compared to commercially insured women (OR = 1.865, *P* < .001).

#### Education

In the last message model, high school/technical school and undergraduate education levels were associated with higher odds of attendance than postgraduate education (high school OR = 1.609, *P* < .001; undergraduate OR = 1.331, *P* = .028).

## Discussion

This study evaluated an email-based DHI that incorporated reinforcement learning and COM-B model-aligned behavioral science strategies to nudge mammogram scheduling and attendance among women overdue for screening and explored whether demographic characteristics moderated messaging effects. Over 2/3 of women engaged with at least 1 email, and among those who engaged, approximately 24% scheduled and 20% attended a mammogram. These rates are consistent with prior messaging interventions targeting breast cancer screenings.^17^^,^^20–22^ For instance, in 1 study, 17.8% of overdue patients (ie, not screened in >18 months) attended a mammogram after receiving a reminder letter.^20^ More recent research reported that 11.2% of overdue women (ie, not screened in the past 2 years) attended mammograms following a mailed reminder letter. Screening rates were not significantly different (10.8%-13.8%) when economic incentives, motivational videos, or access to a scheduling hotline were added to reminder letters.^21^ Other work found that 10.3% of overdue patients (ie, not screened in the past 2 years) scheduled a mammogram appointment after receiving a text-based reminder compared to 6.2% receiving usual care.^22^ Beyond demonstrating feasibility and engagement, these findings extend prior COM-B–informed work by showing that the type and sequencing of behavioral strategies matter in real-world preventive care contexts, particularly among populations already overdue for screening. Specifically, the results suggest that psychological and procedural capability may play a central role in shaping action, even when access and opportunity supports are present, and that delivering capability-focused content at critical early and late touchpoints may be particularly impactful for translating scheduling to completed screenings.

Overall, messages addressing capability barriers (eg, skills, knowledge) were most consistently effective for supporting screening behaviors, whereas messages focused on opportunity barriers (eg, social support, access) were generally less impactful. Capability-focused messages were also more effective than both motivation and opportunity messages for encouraging scheduling, with motivation messages more supportive for scheduling than opportunity content. Attendance outcomes reflected a similar pattern, with both capability and motivation strategies more effective than opportunity messaging at the first intervention touchpoint. At the final touchpoint, capability messages outperformed other content, while the effects of motivation and opportunity messages were similar. Together, these findings suggest that early messages addressing capability or motivation barriers may be especially important for supporting completed screenings, while later outreach works best when capability-focused strategies are prioritized.

Message timing and dose also influenced screening behaviors. In the last message model, both single-message and higher-than-average message doses were associated with lower odds of scheduling and attending a mammogram. Although a single message may be sufficient to prompt screening behaviors for some, minimal intervention exposure may not encourage action among women who are less engaged or hesitant about receiving a mammogram. On the other hand, higher message volumes may contribute to intervention fatigue and diminish engagement. Consistent with this interpretation, exploratory findings indicated that scheduling and attendance peaked at a dose of about 6 messages, independent of message strategy (see Figure S1). This observation suggests that message dose should be carefully ­calibrated to balance sufficient outreach with the risk of overcommunication.

### Health equity outcomes

The impact of COM-B messaging strategies on attendance varied by patient demographics, revealing several opportunities to advance equitable screening. Specifically, messages addressing capability barriers were no longer consistently the most effective approach across demographic subsets. Rather than contradicting the full-sample findings, subgroup outcomes revealed which patients may be underserved by the apparent capability advantage and will be discussed in turn.

Results indicated that early outreach with motivation messages predicted lower odds of attendance in older patients relative to capability-first messaging. Older age was also independently associated with lower odds of attendance following the final message. These findings align with past work showing that although the risk of breast cancer diagnosis is highest in women between the ages of 45-65, older women are more likely to delay, discontinue, or reject screening.^23^ Although the risk of overdiagnosis is higher with age, considerable scientific evidence has demonstrated that the benefits of regular screenings, subserved by advancing technologies, far outweigh the risks of false positives and radiation exposure. While the USPSTF notes insufficient evidence to recommend for or against screening women 75 years and older, they also recognize that clinical decisions involve additional considerations. Specifically, they cite recommendations from the American College of Radiology, American Cancer Society, and American College of Obstetricians and Gynecologists that screening decisions for older women should be individualized to the patient and informed by health status and life expectancy.^1^^,^^24–26^ Taken together, capability-first messaging that underscores the health consequences of missed or delayed screenings and explains the importance of early breast cancer detection may be particularly effective for encouraging screening behaviors in older adults. Providing clear instructions that simplify the logistics of scheduling and attending a mammogram may further improve patient self-efficacy and reduce uncertainty around navigating the visit.

Socioeconomic status and insurance also influenced women’s response to messaging strategies. Low-income women were less likely to be screened than middle-income women following initial outreach but had nearly double the odds of attendance compared to high-income women after the final message. High-income women also had lower odds of attendance than middle-income women, regardless of message position. Both low- and high-income women had higher odds of converting with motivation-based message strategies, with high-income women also benefiting more from opportunity messages than capability messages, which could reflect important socioeconomic differences across factors such as screening anxiety, access, and social support. For example, higher-income patients may benefit from opportunity messaging that draws on support from family, friends, and colleagues, encourages positive comparison to peers or “women like you” who receive screenings, or highlights the convenience and ease of scheduling preventive care for breast health. Messages that reduce concerns about safety, risk, and discrimination may be effective for both low- and high-income women by reassuring patients that they will be treated with care and respect during screening.

Motivation-focused messages also appeared to be more beneficial for women with noncommercial insurance. Although these women had 46% lower odds of attending a mammogram than women with commercial insurance following the first message, moderation analyses indicated that when the final email emphasized motivation rather than capability, women with noncommercial insurance had higher odds of attendance. Prior research shows that uninsured women and those covered by Medicare or Medicaid are more likely to be diagnosed with later-stage breast cancer and experience poorer survival outcomes compared to women with private insurance,^27^ underscoring the importance of early detection as a public health priority. Reassuring messages that focus on reducing fear of discrimination or stigma by motivating patients to identify and compare pros (eg, remaining healthy) and cons (eg, anxiety) of mammograms can help bring clarity to screening decisions and reinforce the patient’s agency in managing their health. Additionally, goal setting from the angle of self-care could be used as a motivational strategy to encourage patients to book their screening early to minimize conflicts with work, caregiving, or other daily responsibilities.

Lastly, although race and education did not moderate the effects of COM-B message content, both were independently associated with attendance. Racially underrepresented women had 47% higher odds of attendance than White women in the last message model, suggesting that tailored digital outreach may help mitigate systemic barriers that contribute to disparities in healthcare, such as minimal access to care. We also found that women with a high school or technical school education had 61% higher odds of attendance and those with an undergraduate education had 33% higher odds of attendance relative to patients with a postgraduate education. These results contrast with other work showing that higher education or non-minority race predicts better health engagement and suggest that more personalized messaging approaches may resonate differently for these demographic subgroups. Sample composition may have contributed to these patterns, as a relatively small percentage of women held postgraduate degrees (15%) or were racially underrepresented (12%). Women with lower education or from underrepresented racial groups may have also faced greater screening constraints that the intervention successfully addressed. The barriers addressed may have been less salient for groups with higher education or from racial backgrounds that have historically faced fewer obstacles to accessing care.

### Strengths and limitations

A key strength of this study is that AI-driven reinforcement learning and behavioral science were combined to deliver a tailored DHI to a large, real-world cohort of women significantly overdue for mammogram screening. This approach is both scalable and responsive to patient barriers. The reinforcement learning algorithm tailored the delivery of human-designed email messages by matching each patient’s demographic profile to the message content most likely to maximize behavioral outcomes, refining selection over time based on observed responses. Because patient demographic characteristics directly informed this matching process, the general capability advantage observed likely reflects the modal patient profile in this sample, whereas the health equity analyses reveal how the algorithm deployed different COM-B strategies for patients with distinct demographic characteristics. Nonuniform screening outcomes across messaging strategies or and patient characteristics suggest that “optimal” DHI implementation decisions are not one-size-fits-all. This finding underscores the value of adaptive, tailored health communications over static, generalized outreach. Understanding and targeting individual barriers to health care decisions is essential for achieving equitable outreach at scale.

Our study has several limitations. First, the DHI targeted mammogram-eligible women who were more than 1 year overdue for screening, a population that is inherently difficult to engage. As a result, interventions delivered to women who are strictly eligible but less overdue may yield more favorable scheduling and attendance outcomes. Second, analyses were limited to patients who opened at least 1 email to discern true intervention effects for those who were exposed to messaging content. Otherwise, the effect of COM-B strategies could not be estimated. However, we report descriptive statistics and comparisons for engaged and unengaged populations. Third, race and health insurance status were analyzed as dichotomous variables to support model stability, given cell size constraints (eg, over 85% of the sample identified as White) and limitations in access to more granular data. As noted previously, available patient EMR data precluded separation of self-pay from other forms of noncommercial coverage. Additionally, the geographic location of the participating health systems in the Midwestern United States may have contributed to imbalanced representation across demographic subgroups. Although aggregating these variables improved model stability, it may obscure meaningful heterogeneity within racial groups and insurance types and should be considered when interpreting results. Fourth, AI-based reinforcement learning selected opportunity messages less frequently than other message types, so corresponding effect estimates may be less stable than those for capability and motivation. Additionally, observed associations between COM-B message content and screening outcomes are inherently confounded by the reinforcement learning algorithm’s adaptive, non-random selection of messages based on patient characteristics and engagement. Consequently, independent effects of COM-B categories on behavior cannot be causally inferred. Fifth, each email message was linked to 1 COM-B category. While multi-component messages can be included in DHIs, we chose singularly focused messages to better isolate the effects of each message type on screening behaviors.

### Suggestions for future research

Future research should evaluate multi-component COM-B messages to determine whether addressing capability, opportunity, and motivation simultaneously produces additive or synergistic effects on screening uptake. Long-term interventions should also be conducted to assess the durability of message efficacy and patient retention over several years. Although we analyzed unique effects of message content at the beginning and end of the behavioral decision process, subsequent work should proceed with directional hypotheses about the impact of intermediate messages on variable participant conversion trajectories (eg, conversions or unsubscribes after 1 message versus 10 or 20). Longitudinal work should also investigate dose-response timelines inclusive of all messages sent to each patient, accounting for nonuniform message composition, sequencing, and conversion trajectories. Including a control group would also enable conclusions about whether messaging using reinforcement learning coupled with behavioral science strategies is superior to no messaging or standard health system messaging. This retrospective study was planned as part of standard-of-care population health outreach, instead of a randomized controlled trial. The DHI was implemented across all eligible patients within each health system and was embedded within preexisting clinical workflows, which made the inclusion of a control group infeasible. As such, the analyses are largely descriptive and exploratory, rather than intended to estimate causal effects. The prospect of withholding outreach raised ethical concerns, as our primary objective was to nudge patients who may benefit most from such health communications (eg, overdue patients). It is also important to note that this study overlapped with the COVID-19 pandemic, which may have affected patient access to mammograms. Diagnostic breast cancer screening decreased by approximately 49% or greater during the pandemic,^28^ with the lowest screening rates observed in minority women.^29^ The total screening deficit was estimated to be 3.9 million people,^30^ and clearing the backlog of delayed care was predicted to take nearly half a year.^31^ Future research should examine whether scheduling and attendance rates remain comparable as overdue patients’ ability to access care is no longer specifically limited by the pandemic.

Subsequent work should also explore how advances in AI can refine and enhance personalization. This DHI used an earlier iteration of AI-driven message sequencing; refined versions have incorporated learnings from the patterns observed here and in prior research from our group. While direct comparisons across health systems or time periods cannot establish causal effects of model updates, future research could investigate whether updated reinforcement learning algorithms produce different engagement and screening patterns, informing methods for optimizing outreach at scale.

## Conclusion

Results from this DHI contribute to the growing field of patient-centered medicine by demonstrating how tailored messaging, guided by reinforcement learning and behavioral science strategies, can promote breast cancer screening. By addressing person-specific barriers mapped to COM-B components (capability, opportunity, and motivation) and applying relevant behavior change techniques, the intervention effectively engaged historically underserved, hard-to-reach populations. These findings underscore the value of strategically sequencing theory-informed messages within adaptive DHIs to target salient behavioral constraints and advance more equitable preventive care delivery.

## Supplementary Material

kaag024_Supplementary_Data

## Transparency statements

(1) This study was not formally registered. (2) The analysis plan was not formally pre-registered. (3) Data from this study are not publicly available due to contractual agreements between Lirio and the health systems where the intervention was implemented. Derived data from this study may be made available (as allowable according to institutional IRB standards) by emailing the corresponding author. (4) Analytic code used to conduct the analyses presented in this study are not available in a public archive. Upon reasonable request, they may be available by emailing the corresponding author. (5) Materials used to conduct the study are not publicly available.
