# Supplementary material for: Retrospective observational study applying reinforcement learning and behavioral science to mammography: insights for equitable preventive care
Source: Ann Behav Med. 2026 May 29;60(1):kaag024. doi: 10.1093/abm/kaag024 (PMC13220756; doi:10.1093/abm/kaag024)
Supplement: kaag024_Supplementary_Data [file kaag024_supplementary_data.docx]

**Supplemental Materials**

Observed screening behaviors by number of messages sent. Each point represents the proportion of patients within a given message count (1-43) who scheduled (orange) or attended (blue) a mammogram. Binning proportions allowed for visualization of trends in screening behaviors across different message volumes. The y-axis shows the within-group proportion of patients, which differs from the overall proportion of screening behaviors across all engaged patients (23.7% scheduled, 20.3% attended). The vertical dashed line indicates the visually observed peak in engagement around six messages.

**Supplemental Figure S1.**

*Exploratory Inspection of Peak Messages Sent Prior to a Scheduled or Attended Mammogram*


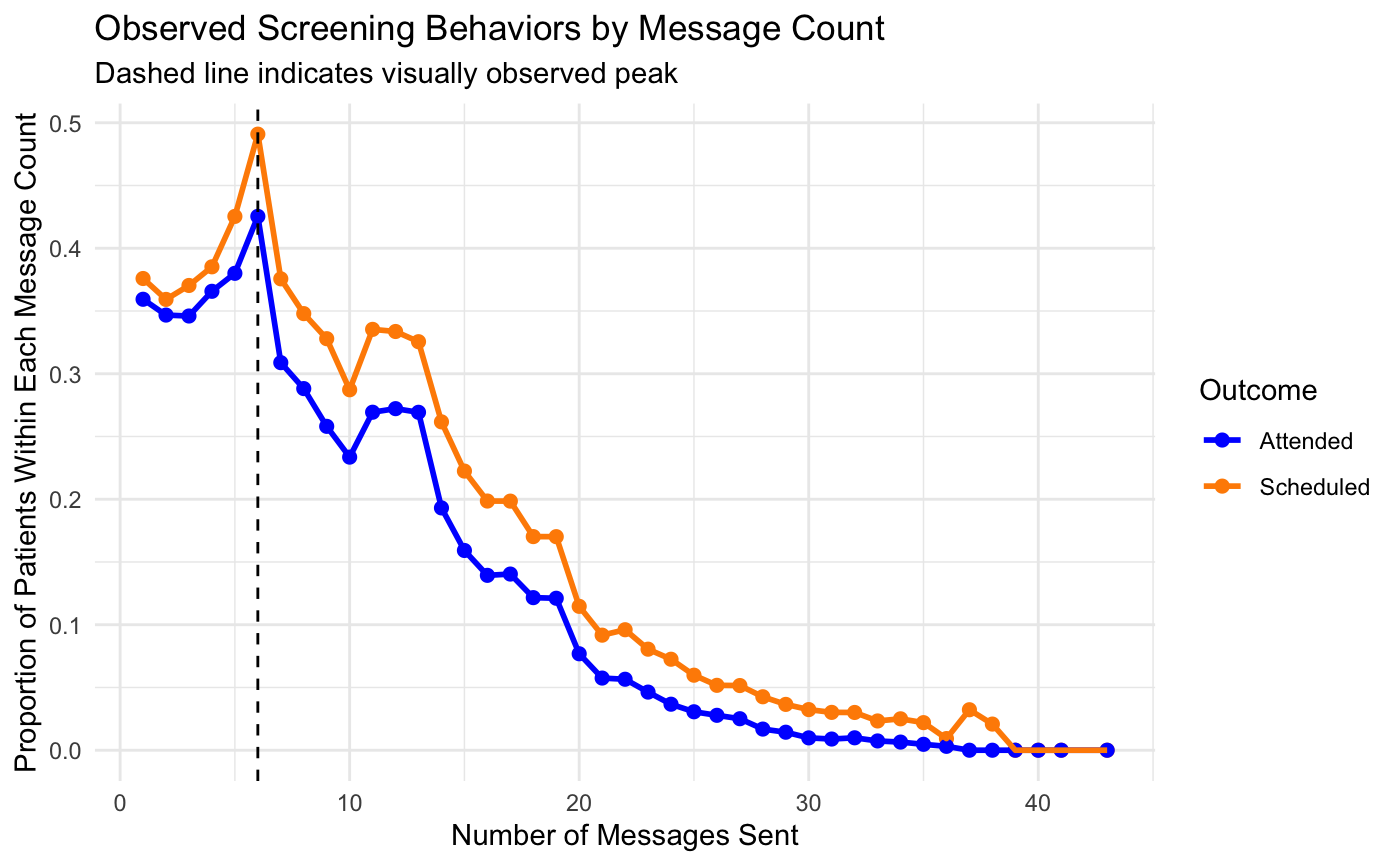


**Supplemental Table S1.**

*Health Equity Analyses: Main Effects and Significant Interactions*

| **Model** |  | **Age** | **Race** | **Income** | | **Insurance** | | | **Education** |
| --- | --- | --- | --- | --- | --- | --- | --- | --- | --- |
| **First Message** | **COM-B comparisons and significant interactions** | *(Mean-centered)* | *White = reference)* | *Middle income = reference* | | *Commercial insurance = reference* | | | *Postgraduate = reference* |
|  |  | **Odds Ratio (95% Confidence Interval), p-value** | | | | | | | |
|  | *Main effect of demographic* | 1.008 (1.000-1.017), p = .05 | Underrepresented: 1.142 (0.945-1.370), p = 0.159 | Low: 0.757 (0.589-0.962), p = .026  High = 0.817 (0.683-0.975), p = .026  Low vs. high: 0.927 (1.774-1.110), p = 0.407 | | Non-commercial: 0.544 (0.474-0.622), p < .001 | | | High school: 1.026 (0.803-1.326), p = .840  Undergraduate: 0.851 (0.653-1.724), p = .239 |
|  | *Motivation vs. Capability* | 1.322 (1.226-1.426), p<.001 | 1.302 (1.200-1.413), p<.001 | 1.398 (1.220-1.606), p<.001 | | 1.288 (1.175-1.415), p<.001 | | | 1.338 (1.036-1.743), p = .03 |
|  | *Opportunity vs Capability* | 1.309 (1.000-1.679), p = .04 | 1.316 (0.989-1.717), p = .051 | 1.836 (1.223-2.660), p = .002 | | 1.131 (0.770-1.604), p = .509 | | | 2.140 (1.023- 4.021), p = .03 |
|  | *Motivation vs Opportunity* | 1.010 (0.772-1.321), p = 0.940 | 1.319 (0.742-1.319), p = 0.942 | 0.761 (0.574-1.009, p = .058 | | 1.139 (0.780-1.663), p = .501 | | | 0.625 (0.301-1.300), p = .209 |
|  | *Significant Interactions* | **Age x Motivation = 0.985 (0.975-0.995), p = .003** | -- | | -- | | -- | -- | |
| **Last Message** | | | | | | | | | |
|  | *Main effects of demographic* | 0.986 (0.979-0.995), p<.001 | Underrepresented: 1.474 (1.218-1.773), p <.001 | Low: 1.088 (0.871-1.35), p = .452  High = 0.545 (0.459-0.645), p = <.001  Low vs. high: 1.996 (1.667-2.391), p <.001 | | Non-commercial: 1.089 (0.951-1.245), p = .215). | | | High school: 1.609 (1.267-2.064), p < .001  Undergraduate: 1.331 (1.036-1.724), p = .028 |
|  | Motivation vs. Capability | 1.221 (1.126-1.325), p<.001 | 1.196 (1.097, 1.305), p<.001 | 1.309 (1.135-1.512), p<.001 | | 1.052 (0.954-1.161), p = 0.312 | | | 1.722 (1.320, 2.261), p<.001 |
|  | Opportunity vs Capability | 2.013 (1.711-2.360), p<.001 | 2.104 (1.769, 2.492), p<.001 | 1.741 (1.361-2.210), p<.001 | | 1.979 (1.555-2.496), p<.001 | | | 2.434 (1.478, 3.874), p<.001 |
|  | Motivation vs Opportunity | 0.607 (0.507-0.726), p<.001 | 0.568 (0.469-0.689), p<.001 | 0.752 (0.567-0.996), p = .047 | | 0.532 (0.411-0.687), p<.001 | | | 0.707 (0.407-1.229), p = .219 |
|  | Significant Interactions | -- | -- | **Low x motivation = 1.347 (1.013-1.795), p = .041**  **High x motivation = 1.439 (1.162-1.784), p < .001**  **High x opportunity = 1.530 (1.070-2.185), p = .019** | | **Non-commercial insurance x motivation = 1.865 (1.577-2.205), p < .001** | | | -- |

*Note.* Statistically significant interactions are shown in bold. Also note that equity analyses adjusting for demographic variables produced different message-level estimates than unadjusted models and observed differences reflect statistical adjustment rather than demographic moderation, except where an interaction reached significance.
